# Falls and Nursing Workload in Hospitalized Older Adults With Delirium: A Controlled Trial of 3D‐Radar‐Based Bed‐Exit Monitoring Versus Contact Mat

**DOI:** 10.1111/jgs.70635

**Published:** 2026-07-31

**Authors:** Tamara Eichenbrenner, Isabella Glaser, Kris Denhaerynck, Vanessa Vater, Diana Fritsch, Valerie Götz, Andreas Fischer, Manuela Pretto, Reto W. Kressig, Wolfgang Hasemann

**Affiliations:** ^1^ University Department of Geriatric Medicine Felix Platter Basel Switzerland; ^2^ University of Basel Basel Switzerland

**Keywords:** bed‐exit monitoring, contactless radar sensing, delirium, in‐hospital falls

## Abstract

**Background:**

Reported in‐hospital fall rates largely rely on subjective incident reporting, and objective verification is rarely used. Evidence on the effectiveness of bed‐exit monitoring systems in patients with delirium is limited, and prospective data on digital contactless systems that do not rely on video‐based monitoring are lacking.

**Methods:**

This prospective, monocentric, non‐randomized controlled study was conducted on a specialized delirium care unit. Analogue bed‐exit monitoring using a contact mat (CareMat) in front of the bed was compared with a digital contactless 3D‐radar bed‐exit monitoring system (QUMEA), which does not generate or record video data. Patients aged ≥ 65 years with delirium were allocated by room availability to analogue or digital bed‐exit surveillance. Bed‐exit‐related falls, daily bed‐exit warnings and nurse presence time were analyzed. Falls were identified using institutional records, electronic health records and system logs. For research purposes only, event‐triggered thermal camera recordings were used for objective fall verification.

**Results:**

In 119 patients contributing 1799 patient days, bed‐exit‐related fall rates were 22.6 per 1000 patient days with CareMat and 11.5 with QUMEA adjusted OR, 3.76 (95% CI, 1.25–11.27). The analogue CareMat device generated more bed‐exit warnings adjusted OR, 1.89 (95% CI, 1.41–2.53) and longer nurse presence times adjusted OR, 1.60 (95% CI, 1.21–2.11).

**Conclusions:**

Under conditions of objective fall verification independent of the bed‐exit monitoring systems, digital contactless 3D‐radar bed‐exit monitoring was associated with lower bed‐exit‐related fall rates and reduced nursing workload compared with an analogue contact mat.

## Summary

- Key Points
  ○ Worldwide in‐hospital fall rates are largely based on subjective incident reporting, and objectively verified fall data are rarely available.
  ○ Evidence supporting the effectiveness of digital bed‐exit monitoring systems for fall prevention remains limited and methodologically inconsistent.
  ○ A contactless three‐dimensional radar‐based bed‐exit monitoring system was associated with fewer bed‐exit‐related falls and reduced bed‐exit‐related nursing workload compared with an analogue contact mat placed in front of the bed.
- Why Does This Paper Matter?
  ○ This study is the first to report objectively verified fall data in hospitalized patients with delirium using a multimodal, system‐independent fall verification approach.

## Graphical abstract

Using objectively verified fall data in hospitalized patients with delirium, a contactless three‐dimensional radar‐based bed‐exit monitoring system was associated with fewer bed‐exit‐related falls and lower bed‐exit‐related nursing workload than an analogue contact mat.

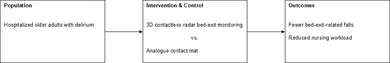

## 1 Background

Patients with delirium are among the most vulnerable in healthcare settings, which necessitates careful consideration of clinical safety and patient rights when evaluating digital monitoring technologies. Delirium is an acute and transient disturbance of attention and awareness, typically accompanied by altered arousal in patients who are not comatose or stuporous [1, 2, 3]. It is associated with increased risk of falls in inpatient and community settings [4, 5, 6].

According to the World Health Organization, “a fall is defined as an event which results in a person coming to rest inadvertently on the ground, floor, or another lower level” [7]. Reported in‐hospital fall rates range from 1.5 per 1000 patient days in Finland [8] to 7.5 in Australia [9], with 0.2–13.3 across US settings [10]. These rates rely on subjective reporting, and only one long‐term care study used objective video verification [11].

Several interventions, including mobility programs, multicomponent strategies, and exercise, have demonstrated fall risk reduction [12], with effects ranging from 10% in meta‐analysis [13] to 30% in individual studies [14]. In addition, analogue bed‐exit warning systems are widely used for fall prevention. However, their effectiveness in reducing falls in patients with delirium remains uncertain. Studies report about delayed alerts, false positives, and lack of fall detection, which have been associated with increased nursing workload [15], contribute to alarm fatigue [16], and elevate the risk of medical errors [17]. Prior studies examined analogue sensors or digital sensor mattresses, reporting limited effectiveness or bias [18, 19]. Furthermore, one systematic review reported a 19% increase in falls among older inpatients using analogue bed‐ or chair‐based sensors [20].

Digital systems may offer a more reliable alternative, as digital sensor mattresses enable objective detection of bed‐exit events when combined with artificial intelligence. However, their effect on in‐room falls remains uncertain due to measurement bias [19].

A further digital development to enable objective detection of bed‐exits and falls is unobtrusive monitoring systems [21]. These are either camera‐based with infrared support for nighttime monitoring or rely solely on infrared cameras. Both approaches may conflict with Swiss and European data protection regulations because video data is considered personal data [22, 23].

In 2020, a specialized delirium care unit (DelirUnit) was established in a Swiss acute care hospital for geriatric medicine to provide enhanced nursing care and continuous monitoring. Given the limitations of analogue bed‐exit systems and the regulatory constraints associated with video‐based monitoring technologies, a more intelligent and law‐compliant solution was sought to overcome these inherent limitations.

A contactless three‐dimensional (3D)‐radar‐based prototype was developed in collaboration with a Swiss technology company (QUMEA, Solothurn, Switzerland). It neither produces video data nor processes personal data and is therefore data‐minimizing. In addition, it uses artificial intelligence to detect bed‐exit patterns and falls. Because QUMEA represents a novel technology, its clinical effects have not yet been systematically evaluated.

This study reports on the effects of the unobtrusive digital 3D‐radar‐based system QUMEA, comprising the bed‐exit warning module (QUMEA BE) and the fall‐detection module (QUMEA FD), compared with the widely used analogue bed‐exit warning system, the contact mat CareMat (BBC Bircher AG, Beringen, Switzerland).

This study aimed to evaluate whether the digital 3D‐radar‐based system is more effective than an analogue contact mat in reducing bed‐exit‐related falls and nursing workload in the DelirUnit.

## 2 Methods

### 2.1 Study Design

This was a monocentric, prospective, non‐randomized, non‐blinded controlled trial on the DelirUnit comparing an analogue and a digital bed‐exit monitoring system (Figures 1 and 2).

**FIGURE 1:**
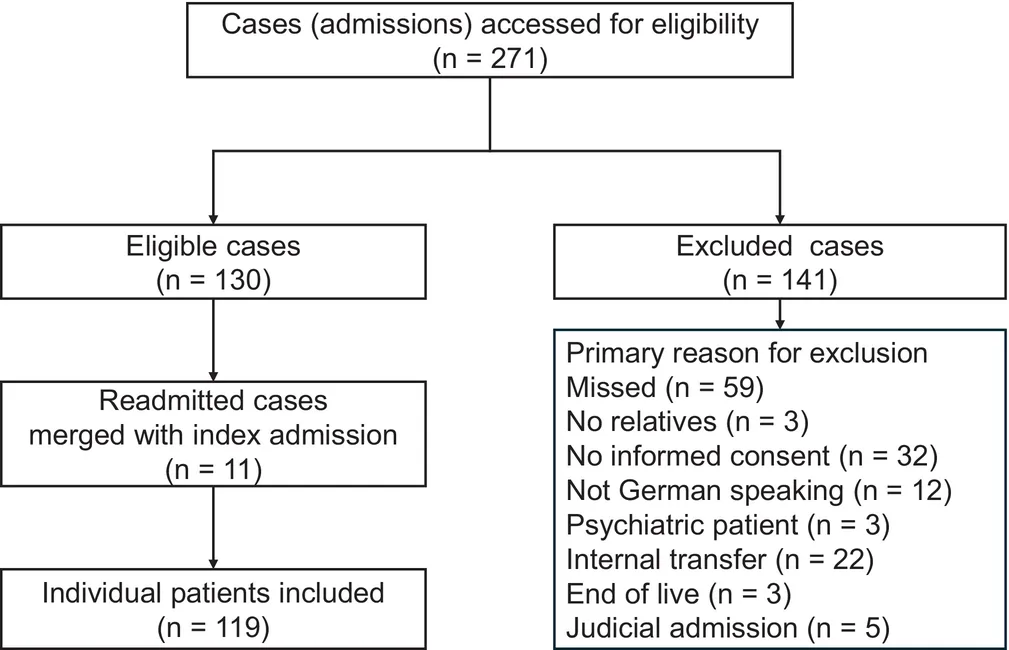
Flow diagram of eligibility assessment and inclusion. A total of 130 eligible cases were identified, corresponding to 119 individual patients; the difference reflects 11 readmissions.

**FIGURE 2:**
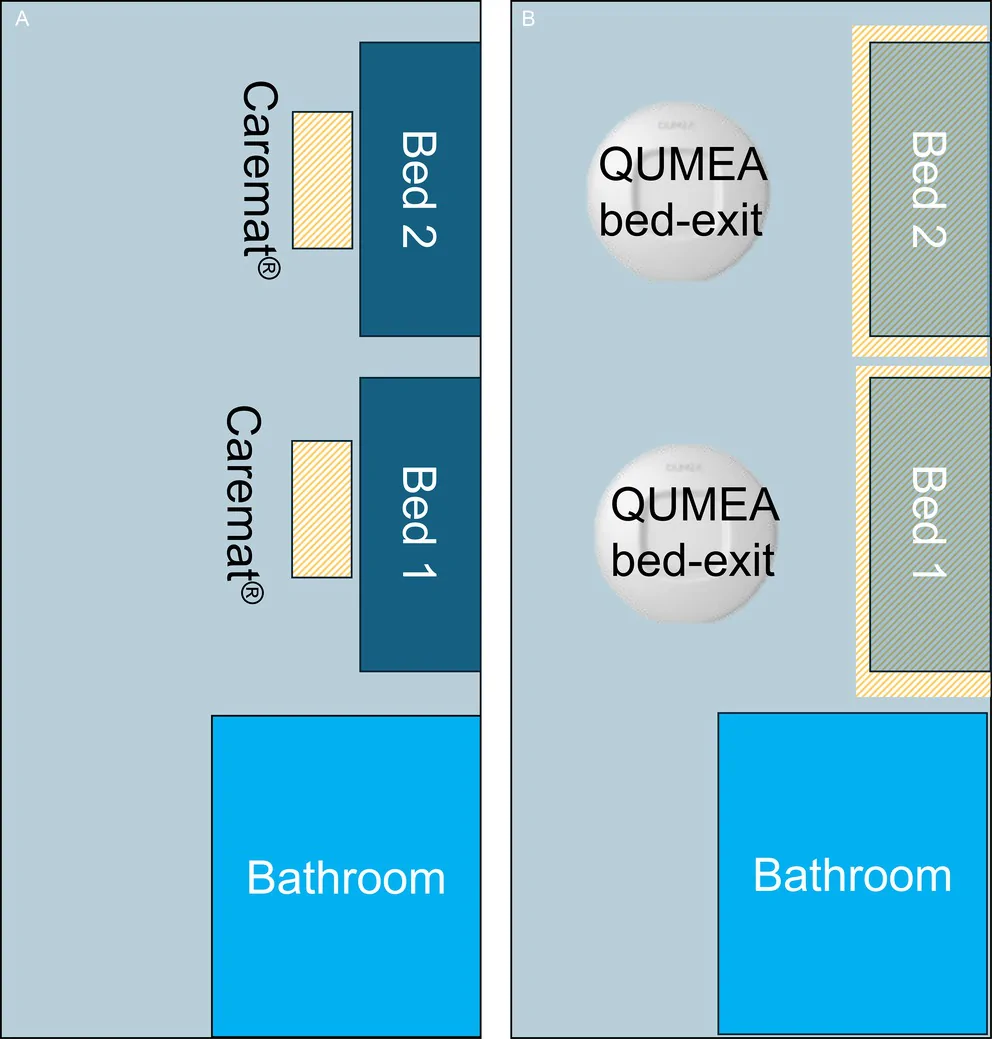
Schematic representation of bed‐exit monitoring systems used in the study. The left panel (A) shows analogue monitoring using a contact mat (CareMat), with the monitored bed‐exit area limited to a surface of 1100 × 700 mm positioned in front of the bed. The right panel (B) illustrates digital contactless bed‐exit monitoring using the QUMEA BE (bed‐exit monitoring) radar system, covering the entire bed area including the lateral sides and the front of the bed.

### 2.2 Setting

The study was conducted on the DelirUnit at the University Department of Geriatric Medicine Felix Platter, Basel, Switzerland. The ward comprises seven double rooms and one single room, admitting up to 12 patients. It is geriatric‐led and staffed by a multidisciplinary team of 22 full‐time equivalents (nurses and assistants), one resident and one senior physician, one advanced practice nurse (APN), and allied health professionals including physiotherapists, occupational therapists, dietitians, social workers, and a chaplain. Facilities include therapy rooms, shared dining areas, and a terrace for therapeutic and recreational activities. Access is badge‐protected to limit traffic and prevent patients from wandering. Because the analogue CareMat system is unable to detect falls, QUMEA FD fall detection was activated in all rooms to ensure a uniform and objective fall‐identification method across both study groups (Figure 3). QUMEA bed‐exit module BE was only activated in intervention rooms and did not affect bed‐exit monitoring in CareMat rooms (Figure 2).

**FIGURE 3:**
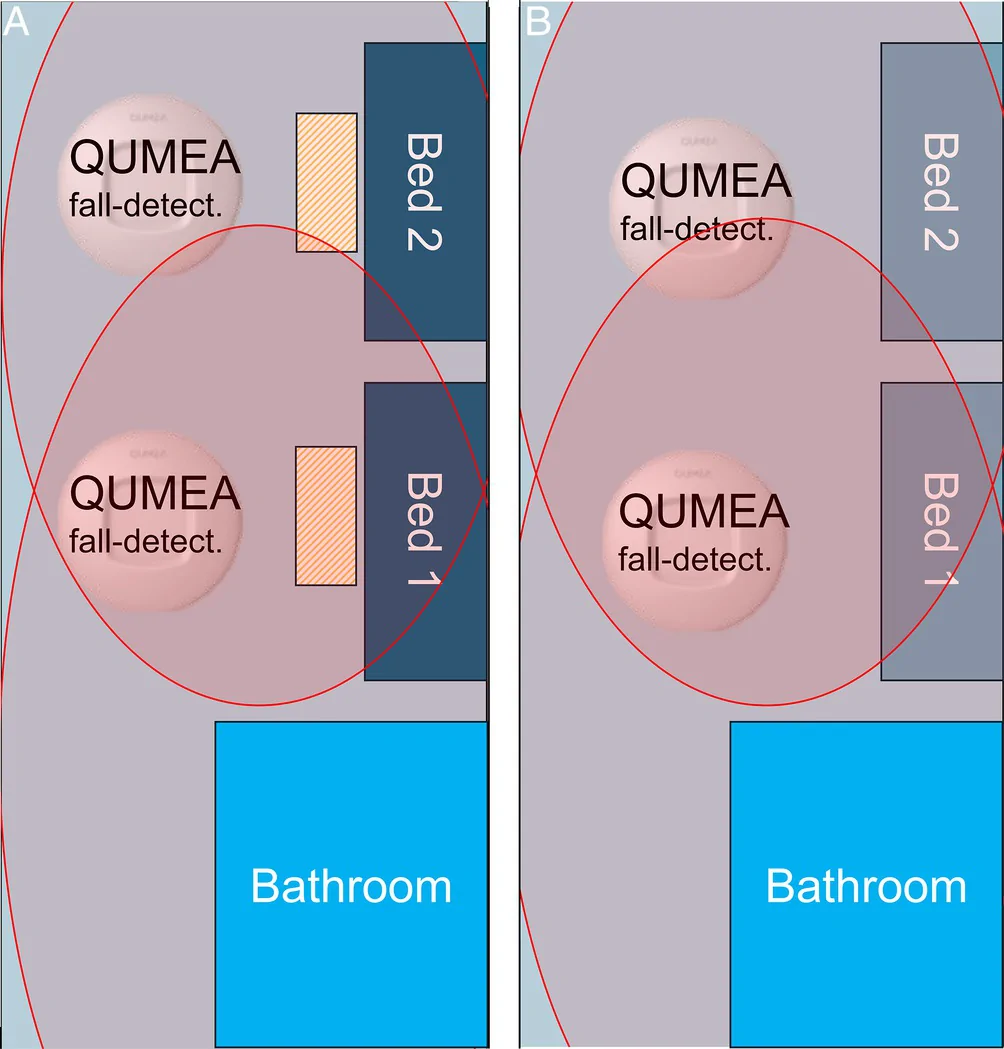
Schematic representation of QUMEA FD (fall detection) in all patient rooms except bathrooms. Two sensors cover the complete room area. FD was active in rooms equipped with CareMat (left panel = A) and in rooms equipped with QUMEA BE (bed‐exit monitoring) (right panel = B).

Delirium care followed a structured non‐pharmacological multicomponent model derived from the Basel Dementia‐Delirium Program, including causal treatment, early mobilization, nutritional and excretion support, sensory optimization, family involvement, and environmental measures to promote orientation and sleep (Data S1) [24, 25].

### 2.3 Participants

Patients aged 65 years or older were eligible for admission to the DelirUnit. The majority of patients (approximately 80%) were referred from surgical, intermediate care, or intensive care units of the University Hospital Basel, including the stroke unit. A few were admitted directly from its emergency department. The remaining patients were referred from home or nursing homes by general practitioners. The main study inclusion criterion was an acutely developed delirium requiring specialized nursing care, enhanced support, and continuous monitoring. Study exclusion criteria were isolation for infectious diseases such as COVID‐19, judicial admission, and inability to obtain proxy consent due to the absence of representatives or language barriers.

### 2.4 Procedures

Once patients had been registered for admission, the APN contacted their relatives, informed them about the ward setting and, if possible, invited them to a meeting to explain the study and obtain written informed consent, as patients with delirium could not consent. Patients were allocated consecutively according to bed availability to rooms equipped either with QUMEA BE or with CareMat contact mats for bed‐exit surveillance. Allocation was determined solely by room availability and not influenced by clinical characteristics or staff decisions. This procedure ensured an unbiased distribution of patients over time. Nursing staff were familiar with both systems, as the QUMEA system prototype had been developed in collaboration with the DelirUnit team.

### 2.5 Intervention: Digital Bed‐Exit Monitoring (QUMEA BE 3D‐Radar‐System)

Bed‐exit monitoring was provided by the QUMEA BE (Figure 2). The system uses a 60‐GHz millimeter‐wave radar sensor and is modular in design, allowing activation of the bed‐exit module only in intervention rooms. Sensors resemble smoke detectors, are mounted on the ceiling and operate contactlessly, ensuring unobtrusiveness and compliance with the data minimization principles of the European General Data Protection Regulation (GDPR) [22] and the Swiss Federal Act on Data Protection (FADP) [23]: no video or personal data, no biometric information, or no body features are captured, processed, or stored.

Data are transmitted securely via LAN to the QUMEA cloud, where neural‐network algorithms distinguish bed‐exit activities from normal movements. Bed‐exit warnings and fall alarms were signaled acoustically and visually through the QUMEA application on nurses' smartphones, enabling classification of false alarms.

### 2.6 Usual Care: Analogue Bed‐Exit Monitoring via Contact Mat

Bed‐exit monitoring in the analogue condition was provided by the CareMat contact mat (Figure 2). The mat (1100 × 700 mm, 9–10 mm height, 8.2 kg) is made of polyurethane, hygienic, fluid resistant, and compatible with disinfectants. A minimum weight of about 10 kg activates the mat, with output via normally open or closed contact.

Signals were transmitted to the QUMEA application, so that nurses perceived no difference between analogue and digital monitoring warnings. The mat is considered obtrusive due to its visible placement and height, which may prompt patients to avoid it or stumble.

### 2.7 Measurements

Falls were identified using four complementary strategies: (1) the institutional fall protocol database, (2) interprofessional documentation in the electronic health records (nurses, therapists, physicians), (3) QUMEA FD database with exact time stamps, and (4) six‐minute infrared video recordings from a separate thermal camera (FLIR Lepton 3.5 micro thermal camera module, Teledyne FLIR LLC, Goleta, CA, USA) triggered by CareMat/QUMEA BE bed‐exit warnings or QUMEA FD fall alarms. These thermal recordings were non‐identifiable, not visible for staff, event‐triggered silhouettes and did not constitute continuous video‐based monitoring. Research assistants independently reviewed all potential events and classified them as true falls or false fall alarms.

The daily number of bed‐exit warnings was extracted from the QUMEA cloud, which provided in this study time stamps for each CareMat or QUMEA event. Nurses' presence time after bed‐exit warnings (i.e., nurse presence time) was measured using time stamps from the Hospicall patient call system (Gummersbach, Germany), documenting room entry and again on leaving. Presence time included the entire in‐room period required to manage the event.

Delirium was assessed with the modified Confusion Assessment Method for the emergency department (mCAM‐ED) [26]. It uses Stillman's Month of the Year Backwards (MOTYB) test to rate attention (range 0–13), higher scores indicating more severe inattention [27] and Hart's logic questions to rate disorganized thinking (range 0–4), higher scores indicating better organized thinking [28]. Furthermore, Kahn's Mental Status Questionnaire is used as a structured interview (range 0–10), with higher scores indicating better mental status [29]. Delirium severity was rated with the Delirium Rating Scale Revised‐98 (DRS‐R‐98) (range 0–39), based on the structured interview of the mCAM‐ED [30]. Higher DRS‐R‐98 indicated more severe delirium. Comorbidity was recorded with the Charlson Comorbidity Index [31]. Functional status was evaluated with the Functional Independence Measure (FIM) [32], and prehospital cognitive status with the 7‐item Informant Questionnaire on Cognitive Decline in the Elderly (IQCODE) [33]. Staffing information was obtained from POLYPOINT PEP (Basel, Switzerland) and from an Excel list of temporary agency staff maintained by the nursing directorate.

### 2.8 Outcome Measures

Primary outcomes were bed‐exit‐related falls occurring in patient rooms under bed‐exit surveillance, the daily number of bed‐exit warnings, and nurses' presence time related to these warnings. Secondary outcomes were the timing of bed‐exit‐related falls across shifts and predictors of warning frequency.

### 2.9 Data Collection

Research assistants collected patient data three times per week (Mondays, Wednesdays, Fridays). They conducted delirium assessments, evaluated falls, and entered all information into a REDCap database (Vanderbilt University, USA). System data from QUMEA for falls, bed‐exits, and the Hospicall patient call system for nurses' presence time were extracted from their respective databases and stored in a Microsoft Access database. Family members were asked to answer the IQCODE and to provide information on prehospital functional status using the FIM.

### 2.10 Statistical Analysis

Descriptive statistics were used to summarize demographic and clinical characteristics. Continuous variables are presented as means with standard deviations and medians with first and third quartiles, categorical variables as counts and percentages. Differences in the daily number of falls between the study groups (QUMEA vs. CareMat) were estimated using random‐intercepts binary logistic regression, while daily number of bed‐exit warnings and daily nurse presence time were analyzed using ordinal logistic regression (random intercepts of shifts nested within patients). Adjusted analyses accounted for the level of (dis‐)organized thinking and correctly memorized words, which turned out to be factors differing between the study groups. Bed‐exit warnings and nurse presence time were not normally distributed and categorized into three groups.

A second set of analyses concerned the same outcome variables but then operationalized per working shift. These models also included candidate correlates of the three outcome variables. Multicollinearity between these correlates was examined. All tests were two‐sided with a significance level of *p* < 0.05. Statistical analyses were performed with SAS (version 9.4, SAS Institute, Cary, NC, USA).

### 2.11 Ethical Considerations

The study was registered at ClinicalTrials.gov (NCT05391334) and approved by the Ethics Committee of Northwestern and Central Switzerland (EKNZ, BASEC‐Nr. 2021–02501).

## 3 Results

Between 1 December 2022 and 10 October 2023, 271 patients were admitted to the DelirUnit. Of these, 119 were included in the study, with 11 patients readmitted once or twice (Figure 1). The median length of stay was 10 days (Q1 = 6.1; Q3 = 16.9). The median age was 85 years (Q1 = 80; Q3 = 89), and 73 patients (61.3%) were male. IQCODE data were available for 84 patients, with a median score of 4.07 (Q1 = 3.43; Q3 = 4.86). Three‐quarters of the patients scored ≥ 3.43, indicating pre‐existing cognitive impairment, whereas the lowest quartile (3.0–3.43) suggested only mild impairment or normal cognition (Table 1). The sample showed substantial heterogeneity in delirium severity and cognitive function (Table S2). Between the first and second assessment, there was a clear trend toward improvement: the median difference was −2 points on the DRS‐R‐98 severity scores, −2 points for MOTYB errors, +2 points for mental status, and + 1 point for disorganized thinking. *Falls* were recorded in 49 of 1799 patient days (27.2 per 1000 patient days), with 29 (59%) occurring in the context of bed‐exit surveillance and 20 (41%) elsewhere on the DelirUnit: bathroom 5 (10%), corridor 5 (10%), shared dining area 4 (8%), and other locations 6 (12%).

**TABLE 1:** Demographic and clinical variables.

	*N*	Mean/%	SD	Median	Q1	Q3	Min	Max
Age	119	84.08	6.80	85.00	80.00	89.00	65.00	98.00
Male	73	61.34						
Female	46	38.66						
Charlson Comorbidity Index	119	3.50	2.30	3.00	2.00	4.00	0.00	11.00
Charlson Comorbidity Index Age adjusted	119	7.45	2.32	7.00	6.00	9.00	2.00	15.00
IQCODE	82	4.12	0.73	4.07	3.43	4.86	3.00	5.00
Total FIM	82	98.91	24.53	106.00	85.00	116.00	20.00	126.00

Abbreviations: FIM, functional independence measure; IQCODE, 7‐item informant questionnaire on cognitive decline in the elderly; Max, highest value; Min, lowest value; Q1, first quartile; Q3, third quartile; SD, standard deviation.

### 3.1 Primary Outcomes

*Fall rates* in the context of bed‐exit surveillance systems were 22.6 per 1000 patient days with CareMat and 11.5 per 1000 patient days with QUMEA, representing higher odds for falls in the CareMat group OR, 3.76 (95% CI, 1.25–11.27; *p* = 0.02) (Table 2).

**TABLE 2:** Primary aims: comparisons of CareMat versus QUMEA.

Outcome	Odds ratio^a^	*p*
Unadjusted		
Daily evaluation of falls^b^	4.02 (1.38–11.74)	0.012
Daily number of bed‐exit warnings^c^	1.83 (1.38–2.43)	< 0.001
Daily nurse presence time^d^	1.55 (1.19–2.03)	0.001
Adjusted^e^		
Daily evaluation of falls	3.76 (1.25–11.27)	0.019
Number of bed‐exit warnings	1.89 (1.41–2.53)	< 0.001
Nurse presence time	1.60 (1.21–2.11)	0.001

^a^ Reference category = QUMEA.

^b^ Binary (no/yes).

^c^ Low 0–3, medium 4–11, high 12–124.

^d^ Low 0–8.99, medium 9.00–31.52, high 31.53–351.06.

^e^ Adjusted for (dis)organized thinking and number of correctly remembered words.

*Bed‐exit warnings* were recorded in 9228 cases during 753 patient days (12,255/1000 days) with the CareMat and 10,209 during 1046 patient days (9760/1000 patient days) with QUMEA. The median number of daily warnings was 8 (Q1 = 3; Q3 = 16) vs. 6 (Q1 = 3; Q3 = 13). CareMat use was associated with a 1.89 higher odds of bed‐exit warnings (95% CI, 1.41–2.53; *p* < 0.001).

*Nurse presence time* was 27,424 min across 770 patient days with the CareMat and 30,379 min across 1083 patient days with QUMEA. The median daily presence time was 23.2 (Q1 = 6.8; Q3 = 47.7) vs. 16.3 (Q1 = 4.5; Q3 = 37.2). CareMat use was associated with 1.60 higher odds for additional minutes per day compared to QUMEA (95% CI, 1.21–2.11; *p* = 0.001).

### 3.2 Secondary Outcomes

Of bed‐exit falls, 41% (*n* = 11) occurred during the night, 37% (*n* = 10) during the day and 22% (*n* = 6) during evening shifts, though these proportions did not differ significantly between shifts. The median number of warnings was 2 during night, 1 during day and 2 during evening shifts, indicating significantly lower numbers during day shifts than night shifts OR, 0.66 (*p* < 0.001) or than evening shifts OR, 0.67 (*p* < 0.001). Also, male patients OR, 1.92 (*p* < 0.001) and those with better cognitive status OR, 1.31 (*p* < 0.001), likely reflecting higher levels of autonomous activity, had more warnings.

Nurse presence time was a median 2.8 min at night, 2.9 during day, and 4.0 during evening shifts, with the evening shift times always significantly longer (Table S3).

## 4 Discussion

This study is the first to report real‐world fall rates in patients with delirium using an objective, multimodal verification process. Falls occurred 3–4 times more often with CareMat than with QUMEA unadjusted OR, 4.02 (*p* = 0.012). Although the reduction with QUMEA was clinically relevant, event numbers were small. In addition, QUMEA rooms showed fewer daily bed‐exit warnings and shorter nurse presence times. Bed‐exit warnings were more frequent during evening and night shifts.

The combined use of the hospital fall database, health care documentation, QUMEA fall notifications and thermal video verification provides a realistic estimation of fall rates. Most international studies are likely to underestimate falls, as incident reporting, retrospective staff recall and incomplete observation commonly miss events [34, 35, 36]. In our setting, thermal video confirmation reduced this bias and supports the validity of the observed fall rates. Compared with fall rates reported in systematic reviews and large observational studies, the rates observed in this study are substantially higher, most likely due to reduced underreporting through objective, multimodal verification [8, 9, 10, 11].

Falls are rare events, and larger powered studies were not feasible because the DelirUnit is the only ward with this concept in Europe. This resulted in wide confidence intervals, reflecting that falls are rare events. Importantly, 41% of all falls occurred outside the surveillance area and were not preventable by either system. Using sitters or physical restraints was neither feasible nor acceptable, as it would have resulted in immobility, where early mobilization remains central to delirium care [37]. For the achievement of rehabilitation aims, that is, the avoidance of bedridden caused sarcopenia to promote patient mobility is imperative in delirium management. This is a major difference of the DelirUnit's concept, compared with usual care units, and was accompanied by rapid improvements in delirium severity over time.

Previous video‐based fall studies had major limitations, including reliance on hospital databases [38], retrospective reporting [39], or incomplete prospective observation [40]. By contrast, in our study, routinely collected data were supplemented by non‐identifiable, event‐triggered thermal recordings independent of staff, enabling confirmation of events without continuous monitoring. In addition, we could show that a digital AI supported radar‐based system was able to reduce the fall rates in patients with delirium, which should be interpreted in the context that the radar‐based system does not rely on optical or visual bed‐exit cues for detection. Our study is, to our knowledge, among the first prospective studies to report fall incidences in the context of digital bed‐exit surveillance systems. Optical‐based systems did not yet report any prospective study‐related effects. Thus, we cannot compare our findings with other digital bed‐exit surveillance systems due to missing evidence.

Contrary to a wider range of potential warning events, particularly based on in‐bed activities, the digital system generated fewer bed‐exit warnings. This reduced nurses' workload, which is particularly important because bed‐exit surveillance may increase workload [15]. The AI‐based algorithm may have better distinguished normal movement from genuine bed‐exit attempts, reducing false alerts and enabling earlier interventions.

Developing bed‐exit surveillance systems that ensure patient rights and comply with Swiss and European data protection laws represents an important consideration in person‐centered care. In contrast, the introduction of a camera‐based bed‐exit system in a Swiss university hospital prompted concerns from cantonal data protection authorities as well as legal and ethics experts, including scholars in health and data protection law. Interestingly, the introduction of such systems is forbidden in France [41].

### 4.1 Limitations

Because room assignment had to follow bed availability to maintain routine operations, cluster randomization was not operationally feasible. Beds vacated in intervention or control rooms had to be reassigned on a first‐come, first‐served basis to maintain routine operations, preventing patient‐level allocation and precluding an RCT design. Shift‐level associations with staffing composition and cognitive variables were exploratory secondary findings and not powered for detailed interpretation.

## 5 Conclusions

The digital bed‐exit warning system QUMEA showed a clinically relevant reduction in bed‐exit‐related falls and fewer bed‐exit warnings and shorter nurse presence times compared with the analogue contact mat CareMat. Our results demonstrate that patient safety and data protection don't need to be conflicting.

## Author Contributions

T.E., R.W.K., and W.H. conceived the study. T.E., V.V., D.F., V.G., and W.H. collected the data. K.D. performed the statistical analyses. All authors contributed to the interpretation of the data and critically revised the manuscript. All authors approved the final version and take responsibility for its content.

## Funding

This work was supported by Velux Stiftung, 1784.

## Ethics Statement

The study was registered at ClinicalTrials.gov (NCT05391334) and approved by the Ethics Committee of Northwestern and Central Switzerland (EKNZ, BASEC No. 2021‐02501).

## Conflicts of Interest

The authors declare no conflicts of interest.

## Supporting information


**Data S1:** Delirium care model.
**Table S2:** Delirium severity and cognitive function.
**Table S3:** Secondary aims: comparisons of CareMat versus QUMEA at the level of the working shift, with candidate correlates as covariates.

## Data Availability

Data may be shared on request.
