# Supplementary material for: Falls and Nursing Workload in Hospitalized Older Adults With Delirium: A Controlled Trial of 3D‐Radar‐Based Bed‐Exit Monitoring Versus Contact Mat
Source: J Am Geriatr Soc. 2026 Jul 31;74(10):2931–8. doi: 10.1111/jgs.70635 (PMC13723201; doi:10.1111/jgs.70635)
Supplement: Supplementary file 1 [file JGS-74-2931-s001.pdf]

Supplementary information S1

Delirium care model

The delirium care model of the DelirUnit at the University Department of Geriatric Medicine Felix Platter is an extension of the Basel Dementia-Delirium Program (DemDel).[1,2] The operational concept of the DelirUnit was further developed from the DemDel principles by a multidisciplinary team including senior physicians, an advanced practice nurse (APN), therapists, social workers, and a chaplain. DemDel is the only German-speaking delirium management program that fulfils all 18 categories of interventions summarized from international delirium management guidelines.[3] Care on the DelirUnit focuses on multicomponent interventions structured in seven main modules:

Causal treatment and medication:

- Identification and treatment of underlying causes of delirium (e.g. infection, electrolyte imbalance, medication side effects, pain).
- Discontinuation of newly prescribed neuroleptics from previous hospital stays. If non-pharmacological measures (e.g. aromatherapy) are insufficient, atypical neuroleptics are prescribed for distressing psychotic symptoms.

Mobilization and nutrition:

- Early mobilization and at least ten 30-minute physiotherapy and occupational therapy sessions per week.
- Patients are encouraged to take their meals in the shared dining room.
- Normalization of food intake with targeted nutritional support.

Normalization of urine and bowl excretion:

- Removal of indwelling catheters under ultrasound monitoring (bladder scan) conducted by nurses.
- Laxative treatment for constipation as required.

Providing normality:

- Encouragement to wear personal clothing.

Sensory support:

- Ensuring patients wear their glasses and hearing aids.
- Provision of hearing amplifiers when no device is available.
- Using aromatherapy with standardized essential oil components, applied either by diffuser or hand massage.

Family involvement and biography:

- The APN contacts relatives prior to admission, collects biographical information, and involves family members in care planning.

Communication, environment and sleep promotion:

- Communication follows Flaherty's TADA principle (Tolerate, Anticipate, Don't Agitate).[4]
- Use of low-floor beds (minimum height 21 cm) positioned parallel to the wall where no bed rails are installed. For patients needing more space, a second mattress can be placed in front of the bed.
- Reduction of noise by forwarding bed-exit warnings and fall alarms to smartphones.
- Promotion of sleep through exposure to daylight on the terrace during family visits and therapy sessions.
- Avoidance of nocturnal disturbance by reducing nighttime routines.

References

1. Hasemann W, Tolson D, Godwin J, Spirig R, Frei IA, Kressig RW. A before and after study of a nurse led comprehensive delirium management programme (DemDel) for older acute care inpatients with cognitive impairment. *Int J Nurs Stud*. Jan 2016;53:27-38. doi:10.1016/j.ijnurstu.2015.08.003
2. Weber C, Fierz K, Katapodi M, Hasemann W. An advanced practice nurse-led delirium consultation service reduces delirium severity and length of stay in orthopedic patients: A nonrandomized posttest only evaluation study. *Perspect Psychiatr Care*. Oct 2020;56(4):804-810. doi:10.1111/ppc.12495
3. Eckstein C, Burkhardt H. Multicomponent, nonpharmacological delirium interventions for older inpatients : A scoping review. *Z Gerontol Geriatr*. Nov 2019;52(Suppl 4):229-242. Multikomponente, non-pharmakologische Delirinterventionen fur altere Patientinnen und Patienten : Ein Scoping-Review. doi:10.1007/s00391-019-01627-y
4. Flaherty JH. The Delirium Room: A Restraint-Free Model of Care for Older Hospitalized Patients with Delirium. In: Malone ML, Capezuti EA, Palmer RM, eds. *Geriatrics Models of Care*. Springer International Publishing; 2015:281-285.

Supplementary Table S2. Delirium severity and cognitive function

	First Assessment								Second Assessment							
	**N**	**M**	**SD**	**Q1**	**Q2**	**Q3**	**Min**	**Max**	**N**	**M**	**SD**	**Q1**	**Q2**	**Q3**	**Min**	**Max**
**DRS-R-98**	113	16.9	7	12	17	21	1	34	87	15.3	6.7	10	15[a]	20	2	31
**Hart**	113	3	1.6	1	3	4	0	4	86	3	1.4	2	4[b]	4	0	4
**MSQ**	113	4.2	3.3	1	4	7	0	10	87	4.6	3.4	2	5[b]	8	0	10
**MOTYB**	111	10.7	4.2	9	13	14	1	14	84	10	4.4	7	11[a]	14	1	14

DRS-R-98=Delirium Rating Scale Revised 98: delirium severity; Hart=disorganised thinking; MSQ=Mental Status Questionnaire; MOTYB=Month of the Year Backwards

M=mean; SD=standard deviation; Q1=first quartile, Q2=median; Q3=third quartile; Min=lowest value; Max=highest value

[a] =lower values showing improvement; [b] =higher values showing improvement

The median interval between the informed consent and first assessment was 2 days (Q1=1, Q3=3)

The median interval between the first and second assessment was 2 days (Q1=2, Q3=3)

Supplementary Table S3. Secondary aims: comparisons of CareMat® vs QUMEA at the level of the working shift, with candidate correlates as covariates

	Number of falls [a]		Number of bed-exit warnings [b]		Nurse presence time [c]	
**Predictor**	**Odds ratio**	**p-value**	**Odds ratio**	**p-value**	**Odds ratio**	**p-value**
**CareMat® vs QUMEA (reference)**	1.81 (0.79–4.18)	0.1620	1.39 (1.14–1.68)	0.0009	1.32 (1.12–1.57)	0.0012
**Shift: Day shift vs Night shift**	1.17 (0.34–4.00)	0.8012	0.66 (0.53–0.81)	<.0001	1.02 (0.83–1.24)	0.8638
**Shift: Evening shift vs Night shift**	0.58 (0.16–2.03)	0.3919	0.98 (0.82–1.18)	0.8677	1.20 (1.01–1.43)	0.0402
**Shift: Day shift vs Evening shift**	2.03 (0.63–6.57)	0.2352	0.67 (0.57–0.78)	<.0001	0.85 (0.73–0.99)	0.0394
**Sex (male vs female)**	2.54 (0.85–7.61)	0.0954	1.92 (1.57–2.36)	<.0001	1.55 (1.30–1.85)	<.0001
**Age**	0.93 (0.88–1.00)	0.0349	0.99 (0.97–1.01)	0.1875	0.98 (0.97–0.99)	0.0012
**# FaGE[d]**	1.10 (0.57–2.15)	0.7727	1.01 (0.90–1.13)	0.9217	1.08 (0.96–1.19)	0.2087
**# Nurses, Higher Education Diploma**	0.82 (0.43–1.58)	0.5544	1.00 (0.90–1.10)	0.9446	1.03 (0.94–1.14)	0.5096
**# Pool**	0.84 (0.49–1.47)	0.5451	1.04 (0.95–1.14)	0.3466	1.06 (0.98–1.16)	0.1623
**# Nurse assistants**	0.97 (0.54–1.74)	0.9081	1.01 (0.92–1.11)	0.8093	1.11 (1.02–1.21)	0.0220
**# External**	0.87 (0.42–1.80)	0.7127	1.16 (1.04–1.30)	0.0082	1.19 (1.07–1.32)	0.0013
**# Correct mem. words**	0.15 (0.02–1.13)	0.0653	0.97 (0.79–1.20)	0.8021	0.92 (0.78–1.09)	0.3364

**Organized thinking**	1.24 (0.96–1.61)	0.0957	1.31 (1.20–1.43)	<.0001	1.25 (1.18–1.32)	<.0001

a Binary (no/yes)

b Low 0, medium 1–3, high 4–56

c Low 0, medium 3.61–8.61, high 8.63–213.17

d Qualified healthcare professionals with federal diploma
